# Diet in the Treatment of Epilepsy

**DOI:** 10.3390/nu13030917

**Published:** 2021-03-12

**Authors:** Cara J. Westmark

**Affiliations:** Department of Neurology, University of Wisconsin, Madison, WI 53706, USA; westmark@facstaff.wisc.edu; Tel.: +1-608-262-9730

This Special Issue for *Nutrients* focuses on the effects of diet on brain function with a special emphasis on epileptic disorders. Altering the diet to treat epilepsy dates back to circa 400 B.C., and the classical ketogenic diet has been utilized since the 1920s. Numerous biochemical changes occur in the brain in response to the ketogenic diet, but how the diet works at a cellular and molecular level remains an enigma. Likewise, there is much to be learned regarding how other diets such as the Atkins diet or plant-based diets affect brain function and the development of seizures. As the use of these diets gain popularity, it is of vital importance to understand their acute and long-term effects on the brain as well as identify their bioactive components. Often, patients resort to natural products and dietary interventions as a means to relieve disease symptoms. Unlike pharmaceutical drugs, which must be proven safe and effective for their intended use before marketing, the FDA does not guarantee the biological activity or purity of dietary supplements. Herbal and vitamin products are sold over the counter and can interact with prescription drugs. We invited review and original research articles that described advances in the field of diet and epileptic disorders for this Special Issue entitled, “Diet in the Treatment of Epilepsy: What We Know So Far”. Epilepsy afflicts 0.5–1% of the world population with a lifetime incidence of 1–3%. A third of patients are resistant to pharmacological therapy. Following are two review articles providing a thorough background on the state of the art of ketogenic diet therapies and five original research manuscripts describing recent diet and dietary supplement findings in animal and human models of epilepsy.

Zarnowska reviews the therapeutic use of the ketogenic diet in refractory epilepsy. The classic ketogenic diet, which mimics starvation, has been utilized for a century to treat epilepsy [1]. The key feature of ketogenic diet therapy is that energy is derived from fat instead of carbohydrates. The macronutrient content consists of high fat, low carbohydrate and sufficient protein for growth. The author reviews the history of the classic ketogenic diet, which was first proposed in 1921 to replace starvation. She also covers key metabolic features, forms and unique features of the ketogenic diet and metabolic therapy that differentiate them from anti-seizure medications as well as possible underlying mechanisms, efficacy, tolerability, adverse effects, barriers in a clinical setting and currently unanswered questions. Although the precise mechanism underlying the success of the ketogenic diet is unknown, it is thought to restore energetic and metabolic homeostasis in the brain. Despite long-time use of the ketogenic diet, there is no agreement on what seizure types and syndromes are likely to respond to treatment, the need for introductory fasting, the role of various types of fat, how ketogenic ratios contribute to success or the role of electroencephalography (EEG) changes in evaluating treatment success. Traditionally, ketogenic diet therapy is considered as a last resort treatment option for drug-resistant epilepsy. There has been limited but promising data regarding ketogenic diet efficacy in other disorders. The author raises interesting points regarding the lack of funding for dietary research versus drug development and varied accessibility to foods and clinical expertise dependent on geographical location. As we move toward patient-centered treatment in clinical practice, ketogenic diet therapy offers a well-established example of an effective intervention, albeit with issues and obstacles that need to be addressed to maintain compliance and retention. A team of professionals including a physician, dietician and psychologist are required along with family support and financial resources. As less restrictive ketogenic diets are clinically validated and become standard practice, it will be imperative to establish the required infrastructure to help families meet compliance and retention guidelines.

Verrotti and colleagues review the evidence-based literature in regard to the use of high-fat diets in the treatment of epilepsy with a focus on the classic ketogenic diet as well as some popular high-fat diet variants including the modified Atkins diet, low glycemic index diet and medium-chain triglyceride diet, which are less restrictive and more palatable [2]. The authors discuss possible mechanisms underlying the success of the ketogenic diet including increased ketone bodies, altered neurotransmitter levels and energy metabolism, increased levels of polyunsaturated fatty acids, reduced apoptosis and changes in intestinal microbiota and the production of pro-inflammatory cytokines. They provide detailed charts covering the clinical data pertaining to the efficacy of the ketogenic diet in pediatric refractory epilepsy and infantile spasms as well as discuss randomized controlled clinical trials, meta-analyses and successful use of the ketogenic diet in Dravet syndrome, Doose syndrome, infantile spasms, febrile infection-related epilepsy syndrome and myoclonic status in non-progressive encephalopathy. Safety and tolerability are reviewed including possible long-term effects on the renal system, bone mineral content, blood lipid levels and micronutrients.

Pasca and colleagues contribute a communication article on family experiences regarding classic ketogenic diet managements under acute medical conditions as assessed by a web-based survey [3]. Survey questions covered demographics, epilepsy diagnosis, ketogenic diet treatment history, the reason for emergency ward admission and outcomes. Fifty families participated, of which 66% had used classic ketogenic diet therapy for more than 2 years, 30% had been admitted to an emergency room at least once and 8% had undergone a surgical procedure. The study found that in 75% of cases, blood ketonemia was not monitored during the emergency room admission. In addition, 46% of survey respondents indicated that medical staff did not have a basic knowledge of ketogenic diet therapy. These findings highlight the need for standard operating protocols in emergency settings for patients on ketogenic diet therapy. Best practices should include patients carrying a vade mecum with a summary of their clinical history, dietary indications, therapies and supplemental prescriptions. At the time of emergency room evaluation, the keto-team should be contacted to share a management plan.

Hung and colleagues examined serum acylcarnitine and amino acid profiles in response to ketogenic diet therapy in pediatric drug-resistant epilepsy [4]. Their study adds to the evidence-based literature regarding identification of biochemical markers to monitor the efficacy of ketogenic diet therapy. Their team administered the classic ketogenic diet under a non-fasting gradual initiation protocol to 29 patients. Twenty-two patients (average age 9.3 years) completed the 12 months of therapy where 23% exhibited seizure reduction of less than or equal to 25%, 18% exhibited a reduction of 25–50%, 41% exhibited a reduction of 50–90% and 18% exhibited a reduction greater than 90%. The responder rate (greater than 50% seizure reduction) was 59%, with 14% of subjects being seizure-free at the end of the study. Blood samples were tested for fatty acid and amino acid levels by tandem mass spectrometry at baseline and after 3, 6, 9 and 12 months of ketogenic diet therapy. Overall, the altered metabolite levels were indicative of decreased glucose breakdown with increased ketosis while improved mitochondrial beta-oxidation was associated with a more positive response to ketogenic diet therapy.

Brunner and colleagues tested the efficacy of uridine and exogenous ketone supplements in decreasing bilateral, synchronous, spontaneous spike-wave discharges in Wistar Albino Glaxo/Rijswijk rats (WAG/Rij), a model of human absence epilepsy [5]. After implanting EEG electrodes in the rat brains, the authors treated cohorts of animals with type A1 adenosine receptor antagonist (A1R), A2R antagonist, uridine and exogenous ketone supplements plus medium-chain triglycerides as well as combinations of these chemicals prior to measuring spine-wave discharges. Uridine (1000 mg/kg) significantly suppressed the number of spike-wave discharges, and this anti-epileptic effect was abolished with A1R antagonist and reduced with A2R antagonist. Co-treatment with a suboptimal dose of uridine (250 mg/kg) and ketone supplements plus medium-chain triglycerides significantly decreased the number of slow-wave discharges. These data speak to the therapeutic potential of a well-tolerated drug, uridine, and confirm an A1R-mediated mechanism underlying efficacy. This study also highlights the anti-epileptic effects attained with co-administration of ketone supplements plus medium-chain triglycerides.

Chang and colleagues conducted a prospective, open, cohort, pilot study of the effect and tolerability of add-on multivitamin therapy in patients with intractable focal epilepsy [6]. Specifically, they tested the multivitamin combination of B6 (100 mg), B9 (5 mg), D (1000 IU), E (400 IU) and Q10 (100 mg) in conjunction with anti-epileptic drug therapy in patients with intractable focal epilepsy. Vitamin levels were quantitated at baseline and 1, 3 and 6 months post-treatment. Effectiveness was assessed by patient diaries. The final analysis included 9 men and 17 women, and the primary outcome measure of seizure frequency was significantly reduced from an average of nine to two seizures per month in response to multivitamin supplementation, with 63% of patients exhibiting a greater than 50% reduction in seizure frequency from baseline, 50% showing a greater than 75% reduction and 13% being seizure-free. There were minimal adverse effects including dizziness (3%), insomnia (3%) and skin rashes (7%), although three patients had worse seizures. This study is the first add-on multivitamin therapy in conjunction with anti-epileptic drugs in intractable epilepsy and has large potential to benefit the 30% of patients with epilepsy who have uncontrolled seizures, particularly considering that vitamins are relatively safe in terms of adverse effects compared to anti-epileptic drugs.

Westmark and colleagues contribute a survey-based study to this Special Issue regarding associations between caregiver-reported use of soy-based infant formula and disease comorbidities in their children with fragile X syndrome [7]. Fragile X is a rare developmental disability characterized by intellectual disability, autism and seizures. The authors build upon their prior work demonstrating associations between the consumption of single-source soy-based diets and increased incidence of seizures in mouse models of neurological disease and in autism. Specifically, they utilized the Fragile X Online Registry with Accessible Research Database (FORWARD) as a sampling frame to recruit 199 subjects from eight clinics across the United States who completed a Fragile X Syndrome Nutrition Study survey. The study found a 25% usage rate of soy-based infant formula with significant associations between soy consumption and increased comorbidity of autism, gastrointestinal problems and allergies. Gastrointestinal problems were the major reason for switching to soy-based infant formula. The data suggest that soy-based infant formula is overutilized in fragile X due to gastrointestinal issues in newborns. There is a dearth of knowledge regarding how early-life feeding affects neurological development, particularly as regards neurodevelopmental disorders. Soy-based infant formulas contain high levels of phytoestrogens and other bioactive components of which the neurological effects largely remain unknown and understudied. Fragile X is not included in the newborn screening panel because a disease-specific intervention is not currently available. Further study of early-life feeding and its effects on neurological development including seizures in fragile X syndrome could provide justification for the implementation of well-validated newborn screening protocols in this population.

Overall, these studies describe exciting new findings in the field of nutrition and epileptic disorder research at preclinical, translational and clinical levels while highlighting the need for increased attention to and resources for the study of dietary effects on neurological outcomes. As evidence-based medicine moves into the realm of personalized medicine, it is imperative to understand the effects of environmental factors including diet on disease outcomes. Please enjoy our collection.
